# Toward the role of social agency in explaining the personalization effect

**DOI:** 10.3389/fpsyg.2024.1405308

**Published:** 2024-11-29

**Authors:** Maike Lindhaus, Julia S. Bolin, Laura Weßeling, Luisa Oest, Stephan Dutke

**Affiliations:** Institute for Psychology in Education, University of Münster, Münster, Germany

**Keywords:** social agency theory, personalization effect, conversational style, task involvement, linguistic personalization

## Abstract

Personalizing written learning materials has been shown to enhance learning compared to conventional text. The aim of the present study was to investigate the role of social agency in explaining the personalization effect. For this purpose, a theory-based scale for measuring social agency was designed including four facets: conversational character, sympathy and emotional connection, explanatory effect, and task involvement. The results of two experiments with *N*_1_ = 66 university and *N*_2_ = 77 high-school students showed that personalized written learning materials compared to non-personalized learning materials were rated higher on the first and partly on the second facet of social agency. However, the personalized materials did not increase learning outcome measures. Consistently, no differences in the task involvement between persons learning with personalized and non-personalized materials were found. Results show that personalization in conversational style alone does not lead to an improvement in learning performance unless other factors intensify task involvement.

## Introduction

Learning from text often occurs in distance learning situations, particularly in recent years, due to the COVID-19 pandemic. One of the main differences of distance learning compared to learning in a shared physical learning environment is the lack of social interaction between learning and teaching persons. This limitation can lead to a decrease in learning motivation (Lin et al., 2020) and subsequently to a decrease in learning achievement (De Paola et al., 2023; Hammerstein et al., 2021).

One way to address this limitation is to personalize the language of written learning materials. Addressing the learner directly, for example, through the use of second-person personal and possessive pronouns (e.g., “your eye “instead of” the eye”) can increase readers’ situational interest and create a sense of direct involvement. Thus, personalization can increase learning motivation and subsequently learning performance (e.g., Dutke et al., 2016). This effect, known as personalization effect, has been studied and replicated several times [e.g., Kurt (2011), Mayer et al. (2004), Moreno and Mayer (2004), and Rey and Steib (2013), for a review see Fiorella and Mayer (2021); for a meta-analysis see Ginns et al. (2013)].

A theoretical framework that is used to explain personalization effects is social agency theory (Mayer and DaPra, 2012; Mayer et al., 2004). According to this theory, learning materials and media can be designed in a way that creates a virtual relationship between learners and the authors of the material. The learner may feel directly addressed similar to being directly involved in a situation in which a “real” communication between learner and author takes place. In this context, the term “personalization” may involve two overlapping meanings. In a conventional view, personalization refers to the impression that the text addresses individual learners whose learning should be initiated and promoted through the text. Additionally, personalization may refer to the impression that the text is the product of a person (the author) whose intention is to initiate and promote learning in the reader. It is assumed that, as a result, conventions of human-to-human communication will apply and the attempt to understand and to follow the communication will increase on the side of the learner. The degree to which a learner feels as an active participant in this communication process is called social agency (Mayer, 2014b).

Social agency theory is used to explain personalization effects (Mayer, 2014b) by hypothesizing that the increased feeling of being an active participant in a quasi-social interaction with the author of the learning materials prompts a deeper processing of the learning contents. However, to the best of our knowledge, this basic assumption of social agency theory and its potentially mediating effect on linguistic personalization have not been explicitly tested. Partially, this may be due to the lack of an adequate measure of social agency for written learning material. The only instrument known to the authors, is the Social Agency Theory Questionnaire (SATQ) by Schroeder et al., 2022, which, however, assesses social agency of pedagogical agents in adaptive training systems. Therefore, this scale is unsuitable to measure social agency as an effect of personalized instructional text.

Preparing the present study, we reviewed research on social agency and related constructs and identified four facets of social agency. In the present study, we describe and pilot a verbal measure of these facets of social agency applicable to instructional text and explored the potential mediation of the personalization effect through social agency.

## Facets of social agency

Social agency and related constructs have been discussed not only in the context of multimedia learning but also in the research on human-computer interaction and the use of virtual agents. A review of the literature in these fields resulted in the identification of four facets of social agency.

*Conversational character* refers to the impression that the author of a learning material is perceived as present and interacting with the learner. Conversational character is based on findings that show that social presence is associated with improved text comprehension in students (Cobb, 2009; Richardson and Lowenthal, 2017). Since the language-related personalization of materials also results in improved text comprehension in various studies, it was reasonable to assume that social presence as a component of social agency theory has an influence on the personalization effect. It has also been shown that people who perceive the author of a text as present develop the feeling of being in a social exchange process (Biocca and Harms, 2002; Mayer et al., 2004). Therefore, items were constructed that measure the extent to which readers of the text perceive the author as present in the sense of instructor social presence and consequently have the feeling of interacting with another person. When the author is perceived as ‘present’ the sense of involvement in social exchange processes is hypothesized to increase, and with it the motivation to follow the communication process and understand its contents (Biocca and Harms, 2002; Cobb, 2009; Mayer et al., 2004; Richardson and Lowenthal, 2017).

*Sympathy and emotional connection* (SE) describe the learner’s perception of the author as a likeable person, equivalent to rating a person in a real social interaction as pleasant. Findings show that texts that address the reader directly leads to greater sympathy from the reader toward the author of the text (De Koning and van der Schoot, 2019). Furthermore, it was found that personalized materials increase the perceived friendliness of the materials (Ginns et al., 2013; Moreno and Mayer, 2004). Sympathy toward the author of the text can therefore be an indication of whether the reader evaluates the author in the context of social psychological scripts, as people would do in a social interaction (Mayer et al., 2004). Consequently, linguistically personalized materials are perceived as more friendly than non-personalized materials (De Koning and van der Schoot, 2019; Ginns et al., 2013; Mayer et al., 2004; Moreno et al., 2001; Moreno and Mayer, 2004). Accordingly, it was assumed that sympathy and emotional connection represent a component of social agency.

*Explanatory effect*: The author is credited with trying to make the text coherent and understandable. According to the cooperation principle (Grice, 1975), if the reader has the feeling of being involved in a social exchange process, they should assume that the author is interested in teaching the reader something and is trying to be informative, accurate, relevant and concise (Mayer et al., 2004). This sense-making process leads to the learner making an effort to understand the meaning of the new information and influences whether the learner can transfer what they have just learned (Atkinson et al., 2005). Social cues (e.g., linguistic personalization) in the material might therefore influence comprehension (Atkinson et al., 2005; Grice, 1975; Mayer, 2014a; Mayer et al., 2004; Schroeder et al., 2022). This construct facet should therefore be used to measure the extent to which the reader perceived the author as motivated to design a comprehensible and explanative text.

*Task involvement* means the willingness to delve deeper into the matters of the text and to develop a deep understanding of what the text is referring to. The assumption is that personalization in conversational style increases the motivation to deal more intensively with the text and promotes the depth of processing and constructive learning processes. Through personalized learning materials, learners are more motivated to understand the learning content and continue to engage with the text (task involvement) (Dutke et al., 2016; Mayer et al., 2004; Moreno et al., 2001; Ginns et al., 2013). According to Social Agency Theory (Mayer, 2014a), this increase of motivation to engage with the materials and deeper processing results from the feeling of being in a social exchange process. Thus, an increased willingness to engage in deeper processing is assumed to be part of social agency.

In our understanding social agency consists of these four facets, each representing and measuring a different aspect of social agency. We expected the four social agency facets both to be separable at the level of EFA (as they measure different facets of the same construct) and moderately intercorrelated at the level of bivariate correlations (as they are part of the same hypothetical construct). Summarized, social agency theory (Mayer et al., 2004) suggests that all four facets of social agency might be triggered by personalized written learning materials. Based on the assumption that feelings of social agency might affect learning behavior, all four facets are reasonable candidates to mediate effects of text personalization on learning outcome.

## Experiment 1

The first experiment investigated the extent to which personalization of learning materials in conversational style has a positive effect on learning performance (measured as text comprehension and transfer performance) and which possible mediating influence social agency has on the personalization effect. Participants learned from text and graphics about human visual perception (with a personalized or non-personalized version of the text) and answered items requiring text comprehension and transfer of information given in the written materials. The non-personalized text did not address the reader directly, whereas in the personalized text definite articles were exchanged by second-person pronouns (“your eye”) so that readers could feel personally addressed. To strengthen the idea of a relationship between readers and author, in one personalized condition, a photo of the fictitious author was added.

To assess the degree of social agency triggered by the personalized vs. non-personalized texts we developed and piloted the social agency scale with four subscales designed to measure the four facets of social agency: conversational character, sympathy and emotional connection, explanatory effect, and task involvement. In the main experiment, we expected participants who studied the personalized text version to score higher in comprehension and transfer items than students who studied the non-personalized text (Hypothesis 1). Since the personalized text was less formal and addressed the participants directly, we expected participants studying the personalized text version to show higher social agency scores than the participants studying the non-personalized text (Hypothesis 2). Additionally, we hypothesized social agency to mediate the personalization effect on learning performance (Hypothesis 3).

### Methods

#### Participants and design

A power analysis using 3.1. G*Power (Faul et al., 2007) to estimate the required sample size for F-tests ANOVA (fixed effects, omnibus, one-way) indicated that three groups with a total of 63 cases were needed to detect an estimated effect size of *d* = 0.42 with an alpha level of 0.05 and a desired power of 0.80 (Cohen, 1992). The expected effect size is based on the results of the meta-analysis by Ginns et al. (2013) and represents the mean of the effects on retention (*d* = 0.30)” and transfer (*d* = 0.54, Ginns et al., 2013, p. 464–465). Averaging these effect sizes, instead of taking the lowest value as a basis for the power analysis, seems justified because studies in other languages than English (among them German) showed a higher mean effect size on retention (*d* = 0.55) than studies conducted English (*d* = 0.25, Ginns et al., 2013, p. 464). Thus, as our study was conducted in German, the effect size we based the power analysis on, is substantially lower than the empirically found effects in studies that can be used for comparison.

Participants were invited by sharing the link to the online study in different German students’ groups on social media (e.g., group chats or study groups). The minimum age for participation was 18 years. Participation was voluntary and the participants were compensated with 10 € for their participation. As we wanted to keep the prior knowledge of the learning content low to avoid confoundation with the effect of personalization on learning outcome, we excluded one person who studied biology.

The final sample included 66 German university students (female = 45; male =21) with an average age of *M* = 23.61 years (*SD* = 5.76). Participants studied different academic disciplines at different German universities (number of participants majoring in different academic disciplines: psychology = 36, chemistry = 10, teaching & education = 3, electronics = 1, computer science = 1, international management = 1, law = 1, sales engineering = 1, mathematics = 1, industrial engineering = 1, not specified = 10) and were randomly assigned to one of three conditions. The control group (*n* = 21) learned with the non-personalized text, experimental group 1 (*n* = 24) received the personalized text without author photo, experimental group 2 (*n* = 21) the personalized text with author photo.

#### Materials and measures

For this study, the same text was used as in Dutke et al. (2016). The participants read a text about the function and structure of the human eye. The text was taken from a German biology textbook for high school students. The non-personalized text consisted of 915 words and a picture showing the components of the human eye in a horizontal section. The text was personalized by exchanging 60 definite articles (“the”) by second-person possessive pronouns (e.g.,” your eye” instead of” the eye”). Minor reformulations assured continuity and coherence [for detailed information see Dutke et al. (2016)]. The content of the learning materials for the three groups was identical.

Eight items about human perception were used to assess the students’ prior knowledge on the function and structure of the human eye. Participants were required to provide a written explanation of terms (e.g., “visual illusion”). Prior to the experiment, a list of criteria for valid explanations was created. Each of the participants’ explanations was rated on a three-point scale (2 = correct description, 1 = partially correct, 0 = incorrect description or no answer). Thus, the highest prior knowledge score that could be reached was 16. Prior knowledge was assessed to ensure that the participants had the same learning prerequisites before the survey and to check that there were no differences in prior knowledge between the groups.

Text comprehension was measured using 13 one-sentence statements about the content of the text. For each statement, the participants rated whether it was true or false. Six statements were false and seven were true; the proportion of false and true statements was unknown to the participants. The items measured the participants’ conceptional understanding of the contents. The learning material contained the information necessary to answer the statements correctly, but this information was formulated differently than in the test statements. Thus, the statements could not be answered solely by remembering the text surface but required text-based inferences. In addition to their true-false decision the participants judged how confident they were to give the correct answers. Combining both judgments resulted in a four-point scale [“I am sure the statement is true”; “I think the statement is true, but I am not sure”; “I think the statement is false, but I am not sure”; “I am sure the statement is false,” see, e.g., Dutke and Barenberg (2015)]. From these data, different performance measures were derived indicating students’ cognitive and metacognitive learning outcome (Barenberg and Dutke, 2013; Dutke and Barenberg, 2015; Barenberg and Dutke, 2019). First, the relative number of correct true-false decisions was computed, which indicated the *correctness* of answering, irrespective of the reported confidence level. This measure served as an indicator of text comprehension. Second, several composite measures of correctness *and* confidence (for formulas see Dutke and Barenberg, 2015, Supplementary Appendix) were computed that reflect different facets of metacognitive monitoring accuracy typically assessed in the field of metacognition (cf. Schraw, 2009; Schraw et al., 2013). The *absolute accuracy* (AC) of the confidence judgments was computed by adding the proportion of correct and confident answers to the proportion of incorrect and unconfident answers. Thus, the AC score reflects the precision of the confidence judgments across all items. The *bias* of the confidence judgments (BS) was computed by subtracting the relative number of correct answers from the relative number of confident answers. Positive values indicate over-confidence, negative values under-confidence. The BS score, however, does not differentiate whether correct and incorrect answers are biased in the same way. Therefore, two conditional probabilities were computed: the *confident-correct probability* (CCP), indicating confidence given the answer is correct, and the *confident-incorrect probability* (CIP), indicating confidence given the answer is incorrect. The difference between both probabilities (CCP − CIP) represents the *discrimination* score (DIS). The higher this difference the more reliably students discriminate between correct and incorrect answers (at the level of confidence). These measures of metacognitive monitoring accuracy were used for exploratory analyses as learning might not only affect cognitive but also metacognitive performance.

Transfer of knowledge was tested using six new problems that could only be solved by the participants applying the information given in the text. A list of criteria for reasonable explanations was created prior to the experiment. Two independent raters scored one point for each criterion met by the participants’ answers. Depending on the complexity of the problem the number of points per transfer task varied between 5 to 8 points. After the first independent rating by two raters, the inter-rater-reliability was high at 89%. Deviating evaluations were settled by discussion. The raters made no use of the option to consult a third rater. The sum of points that could be reached in the transfer items was 41. The percentage of points achieved was used as an indicator of transfer performance.

Social agency was measured using the first version of the social agency scale (Supplementary Appendix A) piloted prior to the first experiment. The scale consists of 16 items, 4 items per facet (conversational character, sympathy and emotional connection, explanatory effect, task involvement). The participants judged the perceived social agency on a 6-point Likert scale, ranging from 1 = *does not apply at all* to 6 = *applies completely*. The mean responses per subscale were used as an indicator of social agency.

Prior to the main experiment, the social agency scale was piloted to investigate its factorial structure and its ability to detect effects of personalized vs. non-personalized text. Data were collected from *N* = 171 university students who studied a scientific text describing the cardiovascular system and the functioning of the human heart. One group was presented with the linguistically personalized text, the other read the non-personalized text variant. The non-personalized version of the text (174 words) did not directly address the reader and was written in a formal style. In the personalized version of the text (172 words), the participants were directly addressed using first- and second-person possessive pronouns (e.g., “your heart” instead of “the heart”). The participants were randomly assigned to the two conditions. After reading the text, participants answered the social agency scale. The internal consistencies (Cronbach’s *α*) of the construct facets conversational character (*α* = 0.89) and task involvement (*α* = 0.84) were very high, the value for sympathy and emotional connection (*α* =0.71) was good and the internal consistency for explanatory effect (*α* = 0.64) was sufficient. Participants who read the personalized text showed significantly higher scores in the facets conversational character and sympathy and emotional connection than the participants who read the non-personalized text (see Supplementary Appendix B). Thus, the social agency scale was able to differentiate between personalized and non-personalized text in two facets of social agency.

#### Procedure

The experiment was designed in Unipark (http://www.unipark.com) and conducted online. The participants were able to access the survey through a link at their chosen time and answer the items in their own pace. First, after activating the link, the participants were presented with the prior knowledge items. Afterwards, they read the text and then filled in the social agency scale and answered the text comprehension and transfer items. During participation the participants were not aware of the two different text conditions. After participation the participants were debriefed and informed about the goals of the study, researchers names and contact information. They had the opportunity to withdraw their consent to participate and to be informed about the study results.

### Results

#### Preparatory analyses

First, we tested whether the photo of the fictitious author enhanced the personalization effect. We found no significant difference between the two experimental groups concerning learning performance [text comprehension: *F*(1, 43) = 0.433, *p* = 0.514; transfer *F*(1, 43) = 0.522, *p* = 0.474] or the social agency facets (conversational character: *F*(1, 43) = 0.200, *p* = 0.657; sympathy and emotional connection: *F*(1, 43) = 2.331, *p* = 0.134; explanatory effect: *F*(1, 43) = 0.173, *p* = 0.679; task involvement: *F*(1, 43) = 0.606, *p* = 0.441). Therefore, in the following analyses, these two groups were combined to one group; homogeneity of variances was given, *F* (1,43) = 0.411, *p* = 0.525.

Second, it was checked whether experimental and control group differed in their prior knowledge. The participants in the control group had a slightly higher mean total prior knowledge score than the experimental group (*M* and *SD* see Table 1). An ANOVA showed that this difference was not significant, *F*(1, 64) = 0.902, *p* = 0.346.

**Table 1:** Means and standard deviations after learning with non-personalized or personalized text in Experiment 1.

Dependent variables	Non-personalized		Personalized	
	*M*	*SD*	*M*	*SD*
Prior knowledge	9.05	3.00	8.27	3.16
Text comprehension	6.90	1.18	7.09	1.29
Transfer	15.28	6.36	13.24	7.46
AC	0.56	0.12	0.57	0.12
BS	0.18	0.18	0.16	0.19
CCP	0.75	0.24	0.75	0.21
CIP	0.68	0.24	0.65	0.26
DIS	0.06	0.24	0.09	0.27

*N = 66.*

AC, absolute accuracy of the confidence judgments; BS, bias of the confidence judgments (positive value: overestimation; negative value: underestimation); CCP, confident-correct probability; CIP, confident-incorrect probability; DIS, discrimination between correctly and incorrectly answered items.

#### Hypothesis testing

We analyzed the text comprehension scores in an ANOVA with type of text (personalized vs. non-personalized) as a between-participants factor. There were no differences between the two groups (*M* and *SD* see Table 1) concerning text comprehension performance, *F*(1, 64) =0.306, *p* = 0.582 and transfer performance, *F*(1, 64) = 1.172, *p* = 0.283. Thus, no personalization effect occurred, and Hypothesis 1 was rejected.

Hypothesis 2 predicted that participants who were directly addressed by the personalized text version scored higher on the social agency scale. We computed four ANOVAs with personalized vs. non-personalized material as the independent variable and the mean scores of the four social agency facets as dependent variables (*M* and *SD* see Table 2). The results revealed that the participants of the experimental group scored higher on the facets conversational character, *F*(1,64) = 21.33, *p* < 0.001, and sympathy and emotional connection, *F*(1,64) = 5.80, *p* = 0.019. In contrast, the mean differences between the text conditions in the facet explanatory effect, *F* (1, 64) = 1.54, *p* = 0.220, and task involvement, *F*(1, 64) = 0.404, *p* = 0.527, were not significant.

**Table 2:** Means and standard deviations of social agency facets after Learning with non-personalized or personalized text in Experiment 1.

Social agency facets	Non-personalized		Personalized	
	*M*	*SD*	*M*	*SD*
Conversational character^a^	2.46	1.07	3.69	0.97
Sympathy and emotional connection^b^	3.51	0.49	3.88	0.61
Explanatory effect	5.16	0.63	4.95	0.68
Task involvement	4.06	1.19	3.87	1.13

*N = 66.*

^a^Mean difference between non-personalized and personalized text, *F*(1,64) = 21.33, *p* < 0.001.

^b^Mean difference between non-personalized and personalized text, *F*(1,64) = 5.80, *p* = 0.019.

The third hypothesis predicted a mediating influence of social agency on the personalization effect. Since we lacked the main effect of personalization on learning performance the mediation analyses were not carried out (Baron and Kenny, 1986).

#### Exploratory analyses

Additionally, to learning outcome measures at the cognitive level, we also tested for personalization effects at the metacognitive level. None of the indicators of metacognitive monitoring showed a difference between the two personalized and the non-personalized text condition; AC, *F*(1, 64) = 0.129, *p* = 0.721; BS, *F*(1,64) = 0.269, *p* = 0.606; CCP, *F*(1,64) = 0.002, *p* = 0.962; CIP, *F*(1,64) =0.225, *p* = 0.637; DIS, *F*(1,64) = 0.262, *p* = 0.610. Thus, personalization did not affect the accuracy of metacognitive monitoring.

As the personalization conditions with and without author photo did not differ in their effect on text comprehension and transfer, we combined both conditions for hypothesis testing. For exploratory reasons, however, we investigated the photo vs. no-photo manipulation with regard to its effect on social agency. An ANOVA showed that there was a significant difference in the score of the social agency facet conversational character between the three groups, *F*(2, 63) = 10.62; *p* = 0.001. The participants in the control group (non-personalized text, *M* = 2.46, *SD* = 1.07) had significantly lower conversational character scores than the participants in the personalized condition (without photo, *M* = 3.75, *SD* = 0.98) (*p* = 0.01) and the personalized condition (with photo, *M* = 3.61, *SD* = 0.98) (*p* = 0.001). However, no significant difference was found between the two personalized conditions (*p* = 0.902). With regard to the social agency facet sympathy and emotional connection, the ANOVA also showed a significant difference between the three groups, *F*(2, 63) = 4.27; *p* = 0.018. The participants in non-personalized condition, *M* = 3.51, *SD* = 0.49, had significantly lower scores than the participants in personalized condition (with photo, *M* = 4.02, *SD* = 0.45, *p* = 0.013). However, there was no significant difference between the control personalized condition (without photo, *M* = 3.75, *SD* = 0.71, *p* = 0.345). Regarding the social agency facets explanatory effect [*F* (2, 63) = 0.850; *p* = 0.432] and task involvement [*F* (2, 63) = 0.493; *p* = 0.613], the ANOVA showed no significant differences between the three groups.

### Discussion

The first hypothesis focused on the personalization effect (Mayer, 2014b) and predicted that the participants learning with the personalized learning material showed significantly higher learning performance (measured as text comprehension and transfer) than participants working with the non-personalized materials. Unexpectedly, the results of the first experiment demonstrated no effects of personalization on text comprehension or transfer, neither at the cognitive nor at the metacognitive level. Thus, in this experiment no effect of linguistic personalization on learning from text emerged. Accordingly, the planned mediation analysis (Hypothesis 3) was not carried out.

The second hypothesis focused on the effect of personalization on the four social agency facets. It was predicted that the group learning with the personalized learning materials should demonstrate higher scores on each social agency facet. The results showed significant differences between the two groups in the facets conversational character and sympathy and emotional connection, with higher values in the personalized condition. Additionally, an exploratory analysis suggested that the photo of the fictious authors may have some influence on the perceived social agency, specifically with regard to the facet sympathy and emotional connection. However, text personalization did not affect the facets explanatory effect and task involvement.

What could be the reasons why the personalization effect was not found? First, lacking validity of the text comprehension and transfer items might be an explanation. However, as the same materials, including the text comprehension and transfer items, were already used in an earlier experiment (Dutke et al., 2016), in which a personalization effect was shown, the problem to demonstrate this effect in the present experiment is probably not due to a lack of item validity. Second, it might be possible that the text, originally authored for school students to read in biology class, was too easy for the sample of university students serving as participants in the present experiment. This interpretation, however, is not substantiated by the data, which did not indicate ceiling effects, neither in the text comprehension nor in the transfer scores. A third reason could be the missing situational relevance of the learning contents. The content of the learning text was unrelated to the participants’ study programs or professional roles. Although the participants were instructed to make serious attempts to understand the learning contents, learning success had no consequences for the participants. Particularly in an online situation this might have limited learning motivation.

A fourth interpretation involves a more differentiated account of the role of social agency. In previous studies (Mayer et al., 2004), social agency was introduced as a theoretical construct hypothesized to explain why a personalized text might help to increase the depth of processing and thus increase learning success. In the present experiment, however, we conceptualized and measured social agency in a more differentiated way, involving four different facets of this construct. Analyzing the scores of these facets showed a differentiated picture of the effects of the experimental manipulation. The personalized learning materials led to higher scores on the social agency facet conversational character and sympathy and emotional connection but not on the remaining facets, explanatory effect and task involvement. That means, personalization, as a feature of the text, was noticed by the participants in that they felt more involved in a virtual interaction with the author of the material than the participants who worked with the non-personalized text. Accordingly, participants in the experimental group felt sympathy and emotional connection toward the author as measured in the facet sympathy and emotional connection. However, the mean scores of the facets explanatory effect and task involvement did not differ between the experimental and the control group. Thus, although participants working with the personalized material felt more involved in a quasi-social interaction (measured by facet conversational character) and partly felt sympathy and emotional connection toward the author of the material (measured by facet sympathy and emotional connection), they (a) did not feel that the author was more engaged in presenting the learning contents in a coherent way (measured by facet explanatory effect) and (b) did not report to process the learning contents in a deeper mode than participants working with the non-personalized material (measured by facet task involvement). This configuration, especially the lack of increased task involvement, could explain why the personalized material did not increase the learning outcome although it made the communication situation more direct and more personal. This explanation is compatible with the idea that the learning situation had only little relevance for the learners. Consequently, personalization did not increase task involvement although it produced a feeling of being addressed.

This interpretation, however, is not unequivocal but open to two different critical views. The first view is that the operationalization chosen in the present experiment was too weak to influence not only the feeling of being addressed but also task involvement. This view would imply that the social agency scale was sensitive enough to detect the effects of personalization, but the experimental situation did not sufficiently emphasize the importance of high learning success and the necessity to process the learning contents in depth. To rule out this explanation, following experiments should include a more meaningful learning situation. The second view is that the experimental learning situation was motivating enough but the social agency scale was insensitive to differences in task involvement or explanatory effect. To rule out the second view, we revised the social agency scale, piloted it again under conditions that allow to investigate its sensitivity regarding all four facets. With the revised social agency scale, Experiment 1 was repeated with a sample to whom the content of the learning material was more relevant (school students).

## Experiment 2

Prior to Experiment 2, the social agency scale was again slightly modified, shortened to a total of 12 items, and piloted again. In this pilot study we also tested whether the relevance of the learning situation influences responses to the items in the task involvement facet. This way, we tried to rule out the interpretation that the task involvement items are not sensitive enough to detect increased task involvement.

Using the revised social agency scale, the first experiment was repeated with a sample of 10th grade school students and the same learning materials and text as in Experiment 1. Experiment 2 was conducted in a school setting and the learning content (function and structure of the human eye) was part of the curriculum. This should assure that the students worked on a learning task that was meaningful to them. As in Experiment 1 we expected students learning with the personalized learning materials to show higher scores in text comprehension and transfer items (Hypothesis 1) and the four social agency facets (Hypothesis 2) than the students studying the non-personalized text. Finally, we expected social agency to mediate the personalization effect on learning performance (Hypothesis 3).

### Methods

#### Participants and design

A power analysis using 3.1. G*Power (Faul et al., 2007) to estimate the required sample size for ANOVA (fixed effects, omnibus, one-way) indicated that 50 cases were needed to test an estimated effect size of *d* = 0.42 with an alpha level of 0.05 and a desired power of 0.80 (Cohen, 1992). The expected effect size is based on the results of the meta-analysis by Ginns et al. (2013) and justified with the same arguments as in Experiment 1. The final sample included *N* = 77 10^th^ grade students (59,7% female; 39% male; 1,3% diverse) of two German high schools.

A two-group design (personalized vs. non-personalized learning material) was employed. Participants were randomly assigned to one of the experimental conditions, *n* = 38 participants read the personalized text and *n* = 39 participants learned with the non-personalized text. Participants were randomly assigned to the two test conditions within each class. Dependent variables were the scores for text comprehension and transfer performance and the four social agency facets. The points scored for prior knowledge were used as a control variable.

#### Materials and measures

For the second experiment, the same non-personalized and personalized texts, prior knowledge test, text comprehension items, and transfer items were used as in Experiment 1. A second experimental group with a personalized text condition and a photo of the fictious author, as used in Experiment 1, was not introduced.

The social agency scale was revised in that some items were reformulated, and the total number of items was reduced to 12 items (see Supplementary Appendix C). The revised social agency scale was piloted with a new sample of *N* = 116 students (89 female, 27 male), participants of two lectures for teacher candidates at the University of Münster. Participants were randomly presented with a personalized or non-personalized version of the text used in the first pilot study. Additionally, in the personalized condition, the participants were informed that the text content was related to the final test in the lecture in the end of the semester. This information was given to emphasize the relevance of the learning material. Participants working with the non-personalized materials received this information immediately after the pilot study. The results of an EFA confirmed the factor assignment of the 12 items. The internal consistencies (Cronbach’s *α*) were high for all four construct facets, conversational character (*α* = 0.90), sympathy and emotional connection (*α* = 0.82), explanatory effect (*α* = 0.82), and task involvement (*α* = 0.83). Comparing the social agency judgments between the personalized and the non-personalized version showed significant differences for the facets conversational character, sympathy and emotional connection, and task involvement with higher values in the personalized condition (for means and standard deviations see Supplementary Appendix D). No significant difference was found between the groups concerning the facet explanatory effect. The pilot results demonstrate that the subscales conversational character, sympathy and emotional connection, and task involvement are suitable to reflect differences in text personalization.

#### Procedure

The study was conducted at school as part of ordinary biology lessons. The general procedure of the experiment was the same as in Experiment 1. The materials (texts, social agency questionnaire and performance test) were given in the same order as in Experiment 1. However, the setting of the study differed. While Experiment 1 was conducted as an online study in which the participants were free to choose the time and place of participation, the data collection in Experiment 2 took place during a lesson in high school with a test instructor present.

Participants were first presented with the prior knowledge test (10 min) and then studied the learning materials on the function of the human eye for 20 min. The test instructor stayed in the classroom for the full data collection, to ensure a quite working environment. After the study phase, the social agency scale was answered and finally the text comprehension and transfer items were answered (50 min). The participants were allowed to use the learning material while answering the items. Participants were debriefed after participation and informed about the experimental manipulation.

### Results

#### Preparatory analyses

First, we examined to what extent the experimental groups differed in terms of their prior knowledge (for *M* and *SD* see Table 3). An ANOVA with text type as the independent variable showed no significant difference, *F*(1, 75) = −1.93, *p* = 0.169.

**Table 3:** Means and standard deviations after learning with non-personalized or personalized text in Experiment 2.

Dependent variables	Non-personalized		Personalized	
	*M*	*SD*	*M*	*SD*
Prior knowledge	5.36	2.21	6.11	2.50
Text comprehension	9.31	1.72	8.97	1.57
Transfer	16.09	5.79	17.36	6.30
AC	0.78	0.13	0.73	0.12
BS	0.06	0.14	0.04	0.16
CCP	0.89	0.15	0.84	0.14
CIP	0.72	0.31	0.64	0.34
DIS	0.16	0.31	0.20	0.34

*N = 77.*

AC, absolute accuracy of the confidence judgments; BS, bias of the confidence judgments (positive value: overestimation; negative value: underestimation), CCP, confident-correct probability; CIP, confident-incorrect probability; DIS, discrimination between correctly and incorrectly answered items.

#### Hypothesis testing

Contrary to the predictions, the results showed no significant differences in learning performance between the experimental conditions – neither for the text comprehension items, *F*(1, 75) = 0.792, *p* = 0.376, nor the transfer items, *F*(1, 75) = 0.844, *p* = 0.361 (*M* and *SD* see Table 3). Thus, no personalization effect occurred, and Hypothesis 1 was rejected.

The second hypothesis predicted that participants who were directly addressed by the personalized text version would score higher on the social agency scale. However the predicted difference was only found for the facet conversational character, *F*(1, 75) = 5.785, *p* = 0.019 (*M* and *SD* see Table 4). Contrary to the hypothesis, no significant differences between the text conditions were found for the remaining three facets (sympathy and emotional connection: *F*(1, 75) = 0.091, *p* = 0.764; explanatory effect: *F*(1, 75) = 0.053, *p* = 0.819; task involvement: *F*(1, 75) = 0.101, *p* = 0.752).

**Table 4:** Means and standard deviations of social agency facets after learning with non-personalized or personalized text in Experiment 2.

Social agency facets	Non-personalized		Personalized	
	*M*	*SD*	*M*	*SD*
Conversational character^a^	2.72	1.15	3.40	1.34
Sympathy and emotional connection	4.50	0.84	4.56	0.83
Explantory effect	4.94	0.79	4.89	0.78
Task involvement	4.15	0.99	4.07	1.25

*N = 77.*

^a^Mean difference between non-personalized and personalized text, *F*(1, 75) = 5.785, *p* = 0.019.

The third hypothesis predicted a mediating influence of social agency on the personalization effect. Since we lacked the main effect of personalization on learning performance the mediation analyses were not carried out.

#### Exploratory analyses

As in Experiment 1, we used the indicated confidence when answering the text comprehension items to calculate indices of metacognitive monitoring. As in Experiment 1, the results showed no difference between the two groups (*M* and *SD* see Table 3) concerning the indices of metacognitive monitoring; AC, *F*(1, 75) = 2.623, *p* = 0.110; BS, *F*(1,75) = 0.596, *p* = 0.442; CCP, *F*(1,75) = 2.067, *p* = 0.155; CIP, *F*(1,75) = 1.084, *p* = 0.301; DIS, *F*(1,75) = 0.264, *p* = 0.609.

### Discussion

The pattern of results resembled very much the pattern found in Experiment 1. First, again the personalization effect on learning did not emerge. Neither text comprehension (at the cognitive and metacognitive level) nor transfer performance were affected by the type of text. As in the discussion of the first experiment, we do not attribute this finding to a lack of item validity because the personalization effect has already been demonstrated with this material (Dutke et al., 2016). As in Experiment 1, no indication of ceiling or bottom effects were found.

The second similarity in the pattern of results in the two experiments relates to the social agency values. In both experiments, the facet conversational character showed higher values in the personalized condition, whereas task involvement did not differ between the conditions. This is consistent with the lack of personalization effects on learning. Task involvement was unaffected by the text type. Thus, it can be speculated that the learning contents in the personalized text condition was not deeper processed as in the non-personalized condition. Consequently, no difference in learning outcome emerged. Nevertheless, the personalized text led to a stronger feeling of being part of a direct communication situation on the side of the learners (facet conversational character). However, this impression did not automatically increase task involvement. The alternative hypothesis that the task involvement items of the social agency scale simply were not sensitive enough for detecting differences in task involvement was ruled out: In the second pilot study we demonstrated that the task involvement values were higher when students worked with the personalized materials *and* were informed that the text contents will be relevant for the end-semester test.

Potential reasons for the lack of the personalization effect in the first experiment might have been (a) the participants’ impression that the learning situation and/or the learning content was not relevant to them and (b) the distance learning situation lacking a shared physical learning environment, and a teaching person present during the test. This situation might have led to low learning motivation (Lin et al., 2020) and subsequently to moderate learning achievement (De Paola et al., 2023; Hammerstein et al., 2021) so that no personalization effect on learning could be observed in Experiment 1. This idea was the reason why we conducted the second experiment in a school context with the learning content as an ordinary part of the curriculum. Comparing learning outcomes between the experiments supports the idea that the students at school (in the second experiment) learned more successfully than the students in the online study (first experiment). The school students obtained higher values in text comprehension and transfer performance.^1^ We conclude that, in the second experiment, the relevance of the learning task was higher than in the less binding online situation of the first experiment. However, even under the conditions of Experiment 2, personalization made no difference regarding learning outcome.

In both experiments, participants felt more involved in a virtual interaction with the author of the material than the participants who worked with the non-personalized material. But different to the first experiment, the participants in the second experiment did not feel more sympathy and emotional connection toward the author than the participants who learned with the non-personalized material. One reason could be the fact that the experimental group in the second experiment did not include a photo of the fictious author. As can be seen from the exploratory results of Experiment 1, a significant difference in the score of the facet sympathy and emotional connection was only found between the control group and the personalized condition with a photo. In the second experiment, such a condition was not introduced which could explain the lack of an effect on the facet sympathy and emotional connection. Another potential reason refers to a difference in the experimental settings. Whereas the first experiment was conducted as an online experiment, the second experiment was carried out at school. In the online situation, the participants had no information about the potential author of the text. Therefore, the style of the text might have a substantial influence on answering the sympathy and emotional connection items. In the second experiment, an individual person instructed the participants in a face-to-face situation, and they might have assumed that the instructor was the author of the text. In this case, the presence of a real person might have influenced responses to sympathy and emotional connection items more than the style of the text. Since the participants of both experimental conditions interacted with the same instructor, the higher influence of the present person might have leveled out the different perceptions of the personalized vs. non-personalized text. This means, although participants working with the personalized material felt more involved in a quasi-social interaction (measured by facet conversational character), they did not feel more sympathy and emotional connection toward the author of the material (measured by facet sympathy and emotional connection). Consequently, the participants in the personalized condition (a) did not feel that the author was more engaged in presenting the learning contents in a coherent way (measured by facet explanatory effect) and (b) did not report to process the learning contents in a deeper mode than participants working with the non-personalized material (measured by facet task involvement). This configuration again could explain why the personalized material did not increase the learning outcome although it made the communication situation more direct and more personal.

## General discussion

Social agency (Mayer, 2014b) was introduced as a hypothetical construct to explain why a personalized text might help to increase the depth of processing and thus increase learning success. So far, however, this hypothesis has not been tested explicitly. Therefore, the present study tested experimentally the role of social agency for the personalization effect and therefore, whether linguistic text personalization affects learning outcome *and* social agency.

In a first step, we analyzed work on social agency and associated constructs in the domains of multimedia learning, distance learning, and human-computer interaction. Based on this analysis, social agency was conceptualized as a multi-faceted construct including four different aspects: (a) conversational character of the learning situation, (b) sympathy and emotional connection with the author of the learning materials, (c) explanatory effect of the materials and (d) involvement in understand the learning contents. Four subscales representing the four facets of social agency were constructed, piloted and revised. Using versions of the social agency scale, we conducted two experiments to test the effect of personalization in conversational style on learning performance and the four social agency facets. We hypothesized that personalization should lead to (a) higher learning performance corroborating the well-established personalization effect and (b) higher values on the social agency subscales. Given such a pattern of results we expected (c) that social agency mediates the personalization effect.

Against expectations, both experiments did not show an effect of text personalization on learning outcome. Next to experiment-specific reasons (as discussed in the previous discussion sections) the role of social agency might be another reason why the personalization effect was not found. The results in both experiments showed that, although participants working with the personalized material felt more involved in a quasi-social interaction (facet conversational character), they (a) did not feel that the author was more engaged in presenting the learning contents in a coherent way (facet explanatory effect) and (b) did not report to process the learning contents in a deeper mode than participants working with the non-personalized material (facet task involvement). This configuration could explain why the personalized material did not increase the learning outcome although it made the communication situation more direct and more personal. Concluding, the assumption that social agency is a unitary construct and as such can explain the emergence of the personalization effect (e.g., Mayer, 2014b) might represent an oversimplification. Alone the reader’s impression of being part of a virtual communication process seems to be insufficient to invoke a personalization effect. Our results suggest that personalization might influence the virtual relation between learners and authors without affecting the way the learning contents is processed – enhancing the learner’s impression of being involved in a quasi-social interaction but leaving learning performance unaffected.

This interpretation can explain why effects of personalization show substantial variance (e.g., Ginns et al., 2013). In some studies, personalization may have increased the impression of being part of a communication situation and task involvement – resulting in enhanced learning. In other studies, such as shown here, only the impression of social involvement was increased without affecting task involvement – yielding no advantage at the level of learning. As in previous studies on personalization, these different facets of social agency were not measured [e.g., Dutke et al. (2016), Kurt (2011), Mayer et al. (2004), Moreno and Mayer (2004), and Rey and Steib (2013); for a review see Fiorella and Mayer (2021); for a meta-analysis see Ginns et al. (2013)], their differential effects could not be disentangled.

### Limitations and future research

Some limitations of the experimental procedures need to be considered. The first experiment included, beyond the control condition with the non-personalized text, two experimental conditions, both employing personalized learning materials – with or without a photo of the fictious author. As the latter manipulation had no effect, these two groups were merged into one group with the effect that the group working with the personalized materials was larger than the control group. As the variances in the compared groups were homogeneous the unequal sample sizes could be tolerated. Nevertheless, this imbalance limits the interpretation of the results and should be avoided in future experiments. However, it should also be noted that in the second experiment experimental and control group were equally large and the pattern of results strikingly resembled the pattern found in Experiment 1. One might discuss whether the experimental groups with and without photo should be treated separately although they had no differential effects on text comprehension and transfer. An exploratory analysis demonstrated that presenting the picture might have influence on the perception of social agency. In the context of the present studies, however, we focused on the general effect of personalization, but it is evident that components that emphasize the existence of a person who is responsible for the learning material (such as an author photo) need to be explored in more detail.

Second, the use of a between-subject design can be discussed. Using a within-subject design would have eliminated between-subject variability, as each subject serves as his or her own control. This increases the accuracy of the results and makes it easier to detect small effects. In the present studies, however, we opted for a between-subjects design as this reduces the working time for the participants. The longer the duration of an experiment the higher is the probability of premature cancellations of participation (in online studies) and the lower is the probability that schools are able to co-operate (in school-related field studies).

Third, the social agency scale constructed for this study needs further validation. Modifications of the initial version based on the pilot studies with *N* = 337 participants ensured the desired four-factor structure and high internal consistencies of the four subscales. First evidence of acceptable sensitivity to differences between personalized and non-personalized texts was also obtained. Nevertheless, more experimental evidence of the scale’s validity in learning context is desirable.

Fourth, the findings of the present study demonstrated that linguistic personalization supports the creation of a virtual relationship between learners and authors of the material as assumed by Mayer et al. (2004). Therefore, learners felt more directly addressed similar to being directly involved in a situation in which a “real” communication between learner and author takes place. This effect has become more relevant the more distance learning is spreading in educational contexts that formerly were dominated by face-to-face interaction because the lack of social interaction between learning and teaching persons was shown to impair learning and learning motivation (De Paola et al., 2023; Hammerstein et al., 2021; Lin et al., 2020). Therefore, future studies on personalization and social agency should focus on distance learning situations and materials typically used in these contexts, such as explanatory videos or modules for self-directed learning.

Last, the results of this study also show that personalization alone does not lead to an improvement in the depth of processing. Although the second experiment was conducted in a school setting conveying curriculum-based learning contents, we could not completely rule out the hypothesis that personalization did not affect learning outcome measures because the contents was not meaningful enough for the participants. Therefore, future studies should further focus on relevant learning situations and personally relevant learning contents to enhance task involvement and measure the influence of personalization on the depth of processing – a postulate that Mayer (e.g., Mayer and Fiorella, 2021) empathetically expressed - not only but also in the context of the personalization principle.

Finally, it should be noted that, in the present studies, personalization was investigated under the perspective of direct effects on learning. Thereby, possible effects on the social climate in class, the relationship between teacher and learner, or behavior indirectly related to learning such as increased class attendance or extended individual learning times were not focused. Thus, the presented results do not necessarily speak against practical application of personalization strategies.

## Conclusion

In conclusion, the findings provide a differentiated view on the role of social agency on the personalization effect. The results show that the readers feel more directly addressed by the linguistic personalization of the learning materials (measured by the social agency facet conversational character), but that this alone does not lead to higher learning performance. Future research should focus on the conditions under which the personalization effect can be replicated considering the possible influence of the different social agency facets on mediating the personalization effect. Future application of personalization in educational settings should focus on ways to enhance not only the feeling of being socially involved but also cognitive involvement in the learning task.

## Data Availability

The raw data supporting the conclusions of this article will be made available by the authors, without undue reservation.

## Footnotes

^1^Text comprehension: *F*(1, 141) = 72.76, *p* < 0.001; transfer performance: *F*(1, 141) = 6.55, *p* = 0.012.
