# Supplementary material for: Toward the role of social agency in explaining the personalization effect
Source: Front Psychol. 2024 Nov 29;15:1405308. doi: 10.3389/fpsyg.2024.1405308 (PMC11637883; doi:10.3389/fpsyg.2024.1405308)
Supplement: Supplementary file 1 [file Presentation_1.pdf]

Appendix A

First Version of Social Agency Scale (as used in Experiment 1)

No.	Item	Facet
1	While reading, I felt like I was in contact with the author.	CC
2	The text made me feel like I was communicating with another person.	CC
3	I do not like the way the author speaks to me.	SE
4	The author of the text has tried to present the text as understandably as possible.	EE
5	I really wanted to read the text to the end.	TI
6	The author encouraged me to engage with the content of the text.	TI
7	The author enjoys teaching others about a topic.	SE
8	The text summarizes the important information.	EE
9	I have a clear idea of what the text describes.	EE
10	The author of the text addressed me personally.	CC
11	I like the way the author speaks to me.	SE
12	The text is written in a friendly tone.	SE
13	I did not feel like I was in contact with the author while reading.	CC
14	The text made me really delve into its contents.	TI
15	The author of the text has not tried to present the text as understandably as possible.	EE
16	I didn't feel like reading the text to the end.	TI

Note. Translated from German. Participants judged the perceived social agency on a 6-point Likert scale, ranging from 1 = *does not apply at all* to 6 = *applies completely*. CC = Conversational character, SE = sympathy and emotional connection, EE = explanatory effect, TI = task involvement.

Appendix B

Pilot Testing of the Social Agency Scale (Version 1, Experiment 1). Results of Unpaired *t*-tests on Differences between Personalized and Non-Personalized Learning Material.

Social agency facet	Non-personalized		Personalized			
	M	*SD*	*M*	*SD*	*t* (169)	*p*
Conversational character	2.67	1.16	3.59	1.18	5.150	<0.001
Sympathy and emotional connection	4.09	0.89	4.40	0.86	2.417	0.017
Explanatory effect	5.11	0.59	4.97	0.69	1.440	0.152
Task involvement	3.98	1.00	3.84	1.01	0.868	0.387

Note. *N* = 171

Appendix C

Second Version of Social Agency Scale as used in Experiment 2

No.	Item	Facet
1	While reading, I felt like I was in contact with the author.	CC
2	The text made me feel like I was communicating with another person.	CC
3	The author made an effort to write this text for me.	SE
4	I really wanted to read the text to the end.	TI
5	The author encouraged me to engage with the content of the text.	TI
6	The text summarizes the important information.	EE
7	I have a clear idea of what the text describes.	EE
8	The author wants me to enjoy reading the text.	SE
9	The author has written a friendly text.	SE
10	I did not feel like I was in contact with the author while reading.	CC
11	The text made me really delve into its contents.	TI
12	I didn't feel like reading the text to the end.	TI

Note. Translated from German. Participants judged the perceived social agency on a 6-point Likert scale, ranging from 1 = does not apply at all to 6 = applies completely. CC =conversational character, SE = sympathy and emotional connection, EE = explanatory effect, TI = task involvement.

Appendix D

Pilot Testing of Social Agency Scale (Version 2, Experiment 2). Results of Unpaired *t*-tests on Differences between Personalized and Non-Personalized Learning Material.

Social agency facet	Non-personalized		Personalized			
	M	*SD*	*M*	*SD*	*t* (113)	*p*
Conversational character	2.32	1.00	3.67	1.40	5.986	<.001
Sympathy and emotional connection	3.64	.93	4.55	1.14	106.32	<.001
Explanatory effect	5.34	.51	5.20	1.09	-.933	.353
Task involvement	3.61	1.06	4.03	1.07	2.101	.038

Note. *N* = 116

Appendix E

Means, standard deviations, and correlations with confidence intervals of social agency facets (final version, pilot testing experiment 2).

Variable	*M*	*SD*	1	2	3	4
1. Conversational character	2.97	1.39				
2. Sympathy and emotional connection	4.08	1.13	.53** [.38;.65]			
3. Explanatory effect	5.27	.84	-.02 [-.12; .17]	.24** [.07; .41]		
4. Task involvement	3.81	1.08	.24* [.06; .40]	.45** [.29; .59]	.33** [.15; .48]	

Note. $N = 116$. M and SD are used to represent mean and standard deviation, respectively. Values in square brackets indicate the 95% confidence interval for each correlation. The confidence interval is a plausible range of population correlations that could have caused the sample correlations (Cumming, 2014). * indicates $p<.05$. **indicates $p<.01$.
